# Myocardial Echinococcosis: Rare Manifestation of a Common Parasitic Disease in Northern Tanzania

**DOI:** 10.7759/cureus.8681

**Published:** 2020-06-18

**Authors:** Ahmed M Jusabani, Clement F Kalambo, Mubashir Jusabani, Salim Surani

**Affiliations:** 1 Radiology, Aga Khan Hospital, Dar es Salaam, TZA; 2 Radiology, Kilimanjaro Christian Medical Center, Kilimanjaro, TZA; 3 Orthopaedics, Kilimanjaro Christian Medical Center, Kilimanjaro, TZA; 4 Internal Medicine, Corpus Christi Medical Center, Corpus Christi, USA; 5 Internal Medicine, University of North Texas, Dallas, USA

**Keywords:** myocardial echinococcosis, hydatid cyst, cardiac mass, lung mass, parasitic cardiac disease, echinococcus

## Abstract

Hydatid (Echinococcal) disease often involves the liver and lungs but in sporadic cases, it can involve cardiac structures. A 24-year-old male was referred with symptoms of cough and shortness of breath and a provisional diagnosis of metastatic disease of unknown primary to the lung, which was based on a chest X-ray (CXR). Incidentally, on echocardiogram, he was found to have right ventricular (RV) and myocardial multiseptated cysts, which were compatible with cardiac echinococcosis, as the patient had multiple bilateral lung cysts as well. Imaging with ultrasound, computed tomography (CT) scan, and magnetic resonance imaging (MRI) has ameliorated the diagnosis of hydatid disease location in various body parts. However, for earlier and accurate diagnosis, a high index of suspicion is required in endemic areas, especially in vulnerable populations such as pastoralists.

## Introduction

Myocardial echinococcosis is a rare disease that constitutes less than 2% of all cases of hydatid disease [1-2]. Hydatid cysts involving the heart are rare and have an indolent course till the small cysts become larger, where there is a high chance of cyst rupture and complications. The overall incidence remains low with an incidence range of 0.5%-2% of the hydatid cases [2-3]. A review of literature in Pubmed and Google Scholar revealed multiple case reports from the Mediterranean region and the Indian sub-continent but, to the best of our knowledge, only a single publication was found from Sub-Saharan Africa (Kenya) [4] and none from Tanzania.

## Case presentation

**Figure 1:**
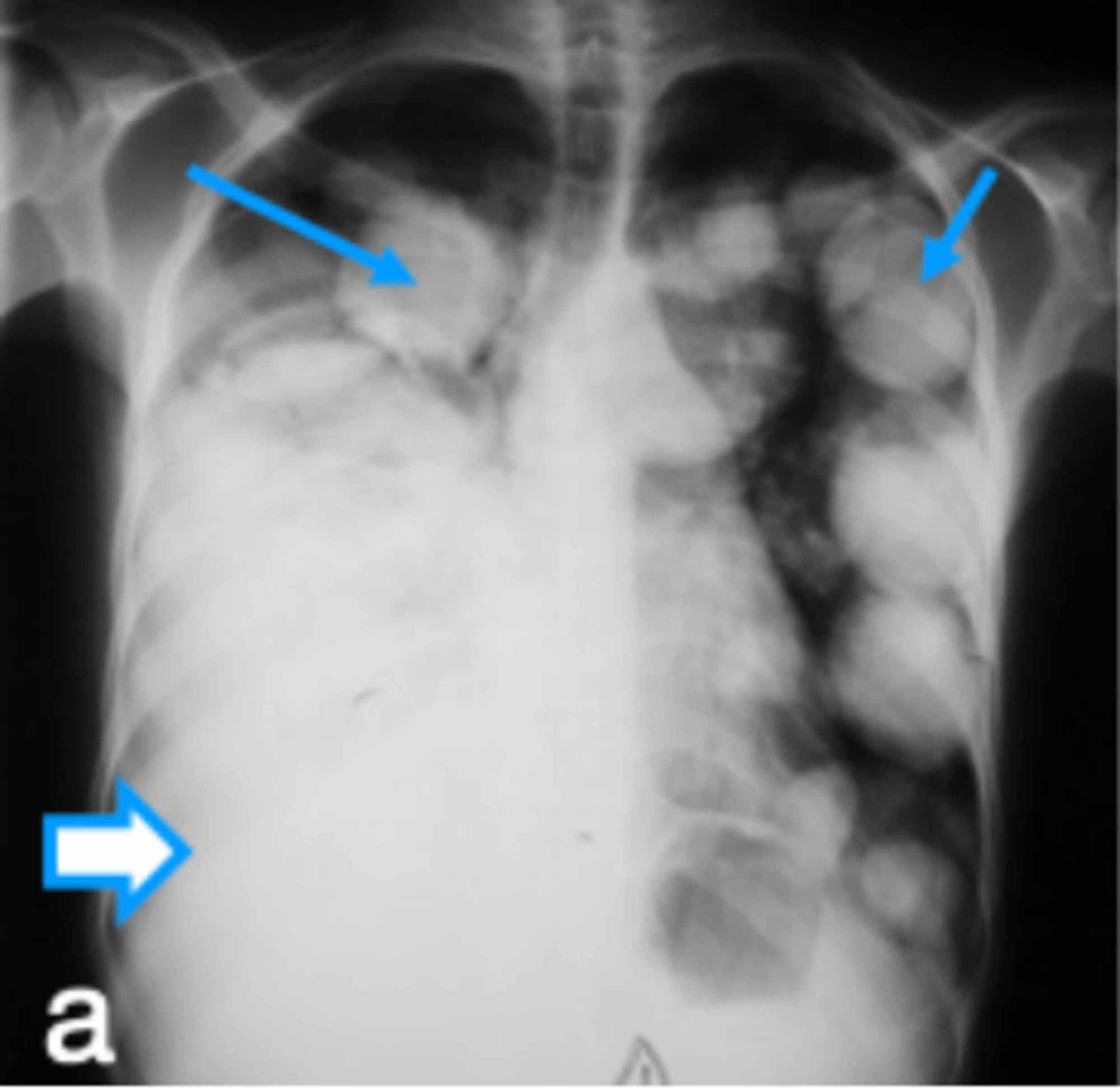
CXR frontal views before drainage of right pleural effusion, revealing multiple bilateral nodules and masses of varying sizes Thin arrows indicate echinococcal nodules and thick arrow indicates right pleural effusion. CXR: chest X-ray

A 24-year-old male patient was referred to Kilimanjaro Christian Medical Centre, a tertiary-level, zonal referral hospital in Northern Tanzania, with cough and difficulty in breathing for eight months, which was gradually worsening and not exacerbated on lying flat. He had a history of intermittent low-grade fevers, excessive night sweats, and weight loss. His referral diagnosis was a metastatic disease of unknown primary based on chest X-ray (CXR) findings (Figure 1).

He belonged to the indigenous Masai tribe, shepherd by profession, single, non-smoker, and non-alcoholic. He was sick looking, febrile, and emaciated with mild pallor. Examination of the respiratory system revealed dullness and reduced air entry on the right side. A review of other systems was normal apart from moderate hepatomegaly.

**Figure 2:**
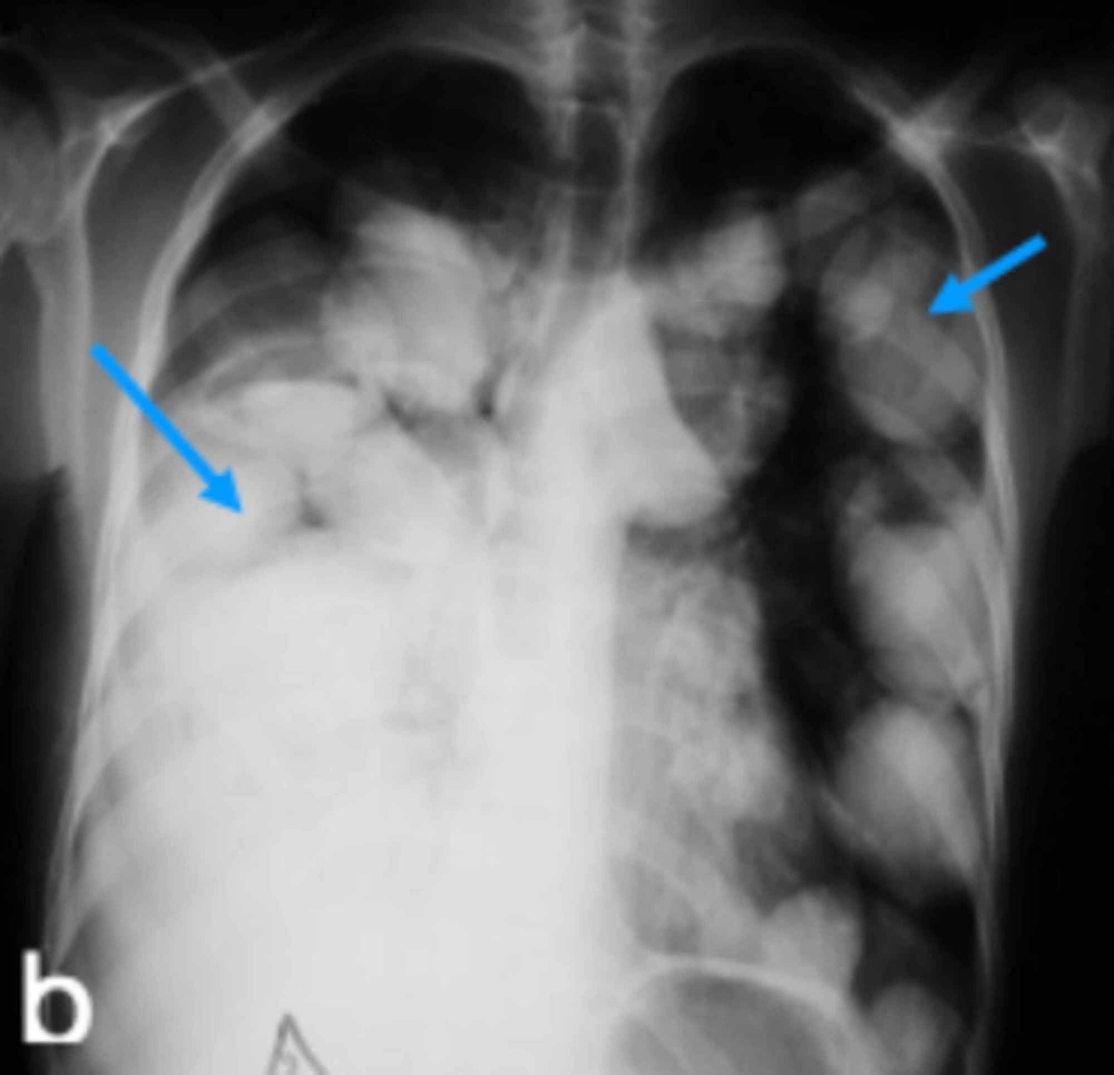
CXR frontal views after drainage of the right pleural effusion revealing multiple bilateral nodules and masses of varying sizes Shorter arrows indicate Echinococcal nodules and the longer arrow indicates decrease in right pleural effusion. CXR: chest X-ray

**Figure 3:**
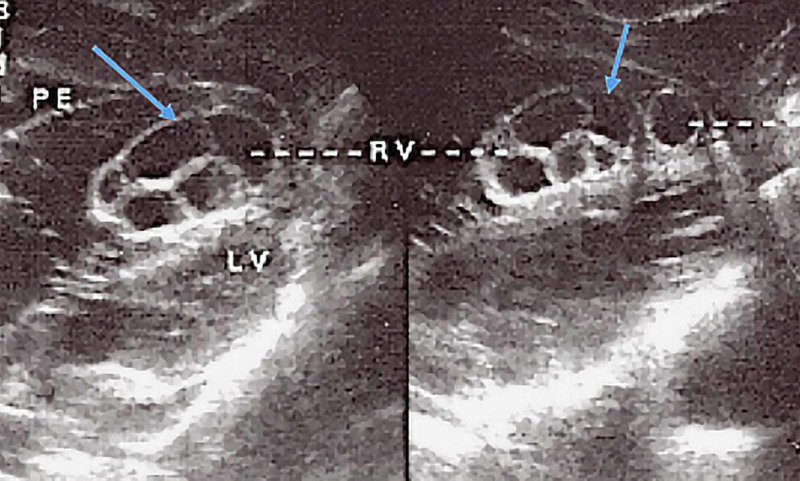
Sub-coastal four-chamber view of the heart revealing RV multivesicular multiseptated cyst (CE2 or type III) and apical unilocular myocardial cyst PE: pleural effusion; LV: left ventricle; RV: right ventricle; M: myocardium

**Figure 4:**
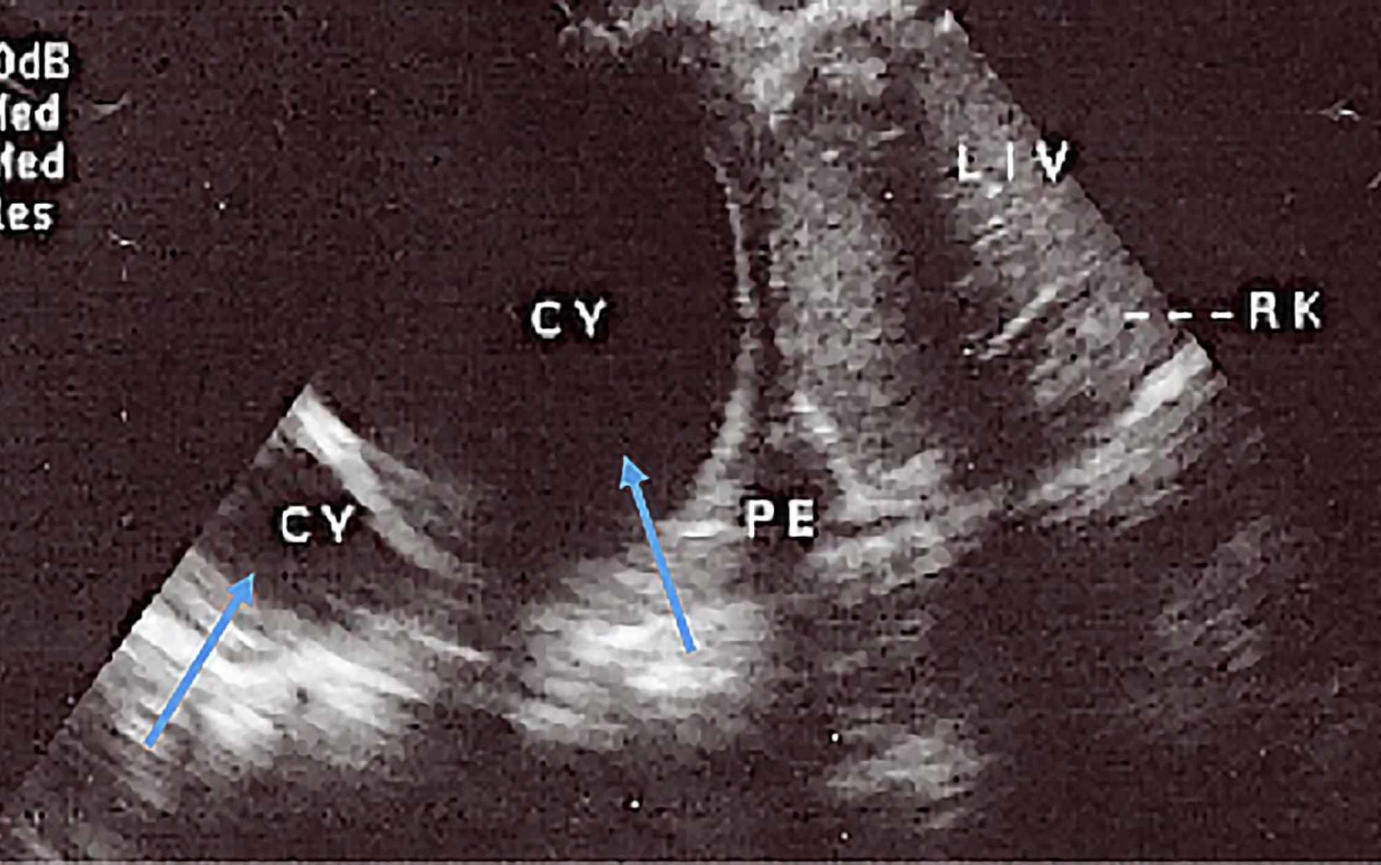
Right subcostal view revealing two unilocular cysts in the right lung lower lobe (CE1 or type I) and trivial pleural effusion PE: pleural effusion; LV: left ventricle; RV: right ventricle; CY: cyst; LIV: liver; RK: right kidney

The initial working diagnosis was pulmonary tuberculosis or lung cancer with right pleural effusion. Laboratory investigations revealed a hemoglobin level of 12 g/dl, white blood cell (WBC) counts, both total and differential, were within normal limits. CXR revealed bilateral multiple nodules and masses of varying sizes and a possible, moderate, right-sided pleural effusion suggestive of metastasis (Figures 1-2). The thoracentesis fluid was exudative, and no malignant cells were seen. Echocardiogram revealed hepatomegaly with no focal lesion, trivial right pleural effusion, and multiple cystic lesions in both lungs. Echocardiogram was performed, which revealed a multivesicular multiseptated cyst (CE2 or type III) and an apical unilocular myocardial cyst in the right ventricle (RV) (Figures 3-4).

The patient was treated with intravenous (IV) fluids and antibiotics and received one unit of blood transfusion. In addition, a right-sided chest tube was inserted and 500 ml of straw-colored fluid was drained. However, after the diagnosis of hydatid disease with pulmonary and cardiac involvement was propounded and the patient was initiated on albendazole 400 mg twice a day for four months. Following the improvement of clinical symptoms, the patient was discharged home in a stable condition; however, he was lost to follow-up.

## Discussion

As far as the life cycle of echinococcus granulosus goes, they live in the intestine of canine animals, especially dogs. Millions of ova are passed on to pastures where sheep and cattle may be grazing. When these animals, and occasionally humans, ingest the ova, the digestive juices dissolve the egg’s external coat, the free hexacanth embryo migrates through the duodenal mucosa and enters the venules and lymphatics. Most of it is filtered by the liver, some by the lungs, and a few may reach distant organs [5].

Hydatid disease commonly occurs in the liver (70%) and lungs (20%) though the extra-hepatic disease does occur in other organs in the body (10%) [6]. It is often difficult to diagnose extra-hepatic echinococcosis, as it is usually not suspected. Symptoms are related to size, location, or ensuing complications of the cyst.

Myocardial Echinococcal disease is not common and tends to remain asymptomatic. The condition for the growth of the parasite is optimal in the left ventricle (LV) [7]. In addition to the RV (15%), the hydatid cysts are also found in the pericardium, pulmonary atrium, left atrial wall, and intraventricular septum [8].

The cardiac complication of hydatid cyst depends on its location within the heart. Conduction disturbances can be seen when the hydatid cyst involves the conduction system, leading to atrioventricular (AV) conduction disturbances and blocks. If the hydatid cysts are in the left ventricle myocardium, it usually tends to be located in the sub-pericardial region, which can rupture into the pericardium causing inflammatory pericarditis. Significant pericardial effusion due to inflammatory pericarditis can cause cardiac tamponade. Anaphylactic shock and sudden cardiac death can also result due to the rupture of the sub-endocardial hydatid cyst [9-10].

Our patient was from the Masai tribe that practices pastoralism in Northern Tanzania, which is endemic to the disease and, therefore, at a higher risk of developing the disease [11-12].

Ultrasound classification of hydatid cysts

Ultrasound is one of the main cost-effective diagnostic imaging modalities for the diagnosis of hydatid cyst. The features of hydatid cyst vary according to the growth stage and associated complications. Radiologists have to be familiar with the appearance from simple cysts to completely solid. classification by Gharbi et al. and the World Health Organization (WHO) classification are the most commonly preferred [13-14]. Computed tomography (CT) scan and magnetic resonance imaging (MRI) play an important role in the diagnostic armamentarium in helping diagnose and recognize the complications of hydatid cysts such as rupture and infection of cysts associated with hydatid disease [15].

## Conclusions

Myocardial echinococcal disease is extremely rare in Sub-Saharan Africa and the clinical presentation is usually insidious. However, a high index of suspicion is required in populations whereby hydatid disease is prevalent and particularly in populations such as pastoralists, as there is always a potential lethal risk of cyst perforation. Ultrasound, CT scans, and MRI have made an earlier diagnosis more probable, therefore, resulting in a reduced incidence of resulting complications and case fatalities.
